# Interpretable Machine Learning for Predicting Neoadjuvant Chemotherapy Response in Breast Cancer Using the Baseline Clinical and Pathological Characteristics

**DOI:** 10.1002/cam4.71221

**Published:** 2025-09-08

**Authors:** Shan Fang, Jun Zhang, Chengyan Han, Mingxiang Kong, Haibo Zhang, Miaochun Zhong, Wuzhen Chen, Hongjun Yuan, Wenjie Xia, Wei Zhang

**Affiliations:** ^1^ Center for Rehabilitation Medicine, Rehabilitation & Sports Medicine Research Institute of Zhejiang Province, Department of Rehabilitation Medicine Zhejiang Provincial People's Hospital (Affiliated People's Hospital), Hangzhou Medical College Hangzhou Zhejiang China; ^2^ Department of Breast Surgery Weifang People's Hospital Weifang Shandong China; ^3^ School of Rehabilitation Hangzhou Medical College Hangzhou Zhejiang China; ^4^ Suzhou Medical College of Soochow University Suzhou Jiangsu China; ^5^ Cancer Center, Department of Radiation Oncology Zhejiang Provincial People's Hospital, Affiliated People's Hospital, Hangzhou Medical College Hangzhou Zhejiang China; ^6^ General Surgery, Cancer Center, Department of Breast Surgery Zhejiang Provincial People's Hospital (Affiliated People's Hospital), Hangzhou Medical College Hangzhou Zhejiang China; ^7^ Department of Breast Surgery (Surgical Oncology), Second Affiliated Hospital Zhejiang University School of Medicine Hangzhou Zhejiang China; ^8^ Geriatric Medicine Center, Department of Endocrinology Zhejiang Provincial People's Hospital, Affiliated People's Hospital, Hangzhou Medical College Hangzhou Zhejiang China

**Keywords:** breast cancer, interpretable machine learning, neoadjuvant chemotherapy, pathological complete response, tumor‐infiltrating lymphocytes

## Abstract

**Background:**

The pathological response to neoadjuvant chemotherapy (NAC) has become a vital prognostic indicator for patients with breast cancer (BC). The newly generated models depended on rather basic imaging and pathology characteristics and did not sufficiently elucidate the importance of the incorporated data. The purpose of this study is to establish and authenticate a machine learning model for predicting the pathological complete response to NAC using baseline clinical and pathological features in BC patients.

**Methods:**

Data were collected from hospitalized BC patients treated with NAC at Zhejiang Provincial People's Hospital between January 2014 and August 2023. The dataset was randomly split, with 70% allocated for model training and 30% for validation. LASSO regression was used to select predictive features. Six ML models—XGBoost, LightGBM, CatBoost, logistic regression, random forest (RF), and support vector machine (SVM)—were developed, with performance assessed using the area under the curve (AUC) and accuracy, precision, recall, F1 score, and Brier score. Clinical benefits were evaluated using decision curve analysis (DCA), and SHapley Additive exPlanation (SHAP) was applied to interpret the features of the optimal ML model.

**Results:**

A total of 303 bc patients treated with NAC were included, with a pCR rate of 29.37% (89/303). Twelve features, such as age, menopausal status, PR, HER2 status, Ki‐67 expression, stromal tumor‐infiltrating lymphocytes (sTILs) et al., were selected for model construction. Among the six models, the CatBoost model demonstrated the best predictive performance, achieving an AUC of 0.853 after Bayesian hyperparameter tuning. SHAP analysis ranked sTILs as the most critical predictive feature. In fivefold cross‐validation, the CatBoost model incorporating sTILs achieved an average AUC of 0.83.

**Conclusions:**

The ML‐based pCR prediction model enables more accurate pCR prediction for BC patients at baseline, aiding in optimizing treatment strategies. Additionally, the interpretable SHAP framework enhances model transparency, fostering clinical trust, and understanding among doctors.

**Abbreviations**
AUC: area under the receiver operating characteristic curve
BC: breast cancer
BMI: body mass index
CatBoost: categorical boosting
CEA: carcinoembryonic antigen
DCA: decision curve analysis
LightGBM: light gradient boosting machine
LR: logistic regression
ML: machine learning
NAC: neoadjuvant chemotherapy
pCR: pathologic complete response
ROC: receiver operating characteristic curve
SHAP: SHapley additive exPlanations
sTILs: stromal tumor infiltrating lymphocytes
SVM: support vector machine
XGBoost: eXtreme gradient boosting

## 1 Introduction

Breast cancer (BC) is one of the most prevalent tumors among women worldwide [1]. While early BC generally has a favorable prognosis, the prognosis remains suboptimal due to the high risk of metastasis [1]. Pathological response to neoadjuvant chemotherapy (NAC) is an essential predictor of survival in BC patients, with pathological complete response (pCR) having a strong, positive link to survival outcomes [2, 3, 4]. Customized post‐neoadjuvant therapy holds promise for enhancing long‐term prognosis in patients who do not achieve pCR [5, 6]. Ethnic, racial, and tumor‐specific characteristics also influence BC outcomes, underscoring the importance of diverse and inclusive datasets in developing accurate predictive models [7, 8].

Recently, studies have proposed artificial intelligence (AI) has shown remarkable potential in advancing breast cancer diagnosis, treatment, and management, and machine learning (ML)‐based models could be used for predicting BC outcomes [9, 10, 11, 12]. These studies underscore the significance of machine learning models in developing accurate predictive models and personalized precision therapies, as compared to traditional approaches. Machine learning can work with complex data sets without specifying complex relationships in a good deal of variables a priori, so it is considered a promising analytical method. However, these previous models had many flaws that require further clarification. First, they relied on relatively simple imaging features and especially few pathological characteristics. Second, they failed to adequately explain the significance of the included features, and their model had relatively low interpretability [9, 10, 11, 12]. Additionally, some incorporated post‐treatment information, which was not suitable for predictive models intended for baseline evaluations. In this study, we aimed to outperform a prior model to predict BC outcomes and enhance the interpretability.

The tumor microenvironment exerts a significant influence on the initiation and progression of tumors. Tumor‐infiltrating lymphocytes (TILs), essential regulators in the tumor microenvironment, are classified into intratumoral and stromal TILs (sTILs) subtypes based on their location in the tumor nest or stroma [13, 14, 15]. Recent research has demonstrated that an immune‐rich microenvironment is associated with improved prognosis in both early and advanced BC. As critical markers of the tumor microenvironment [16, 17, 18], TILs have been identified as significant predictors of response to NAC in BC [19, 20, 21]. Moreover, our previous study highlighted the important role of sTILs in predicting BC outcomes [22]. Building on these findings, we aimed to develop an accurate and promising ML‐based prediction model incorporating various pathological and clinical features to facilitate personalized treatment and improve BC prognosis.

## 2 Materials and Methods

### 2.1 Study Population and Data Source

**FIGURE 1:**
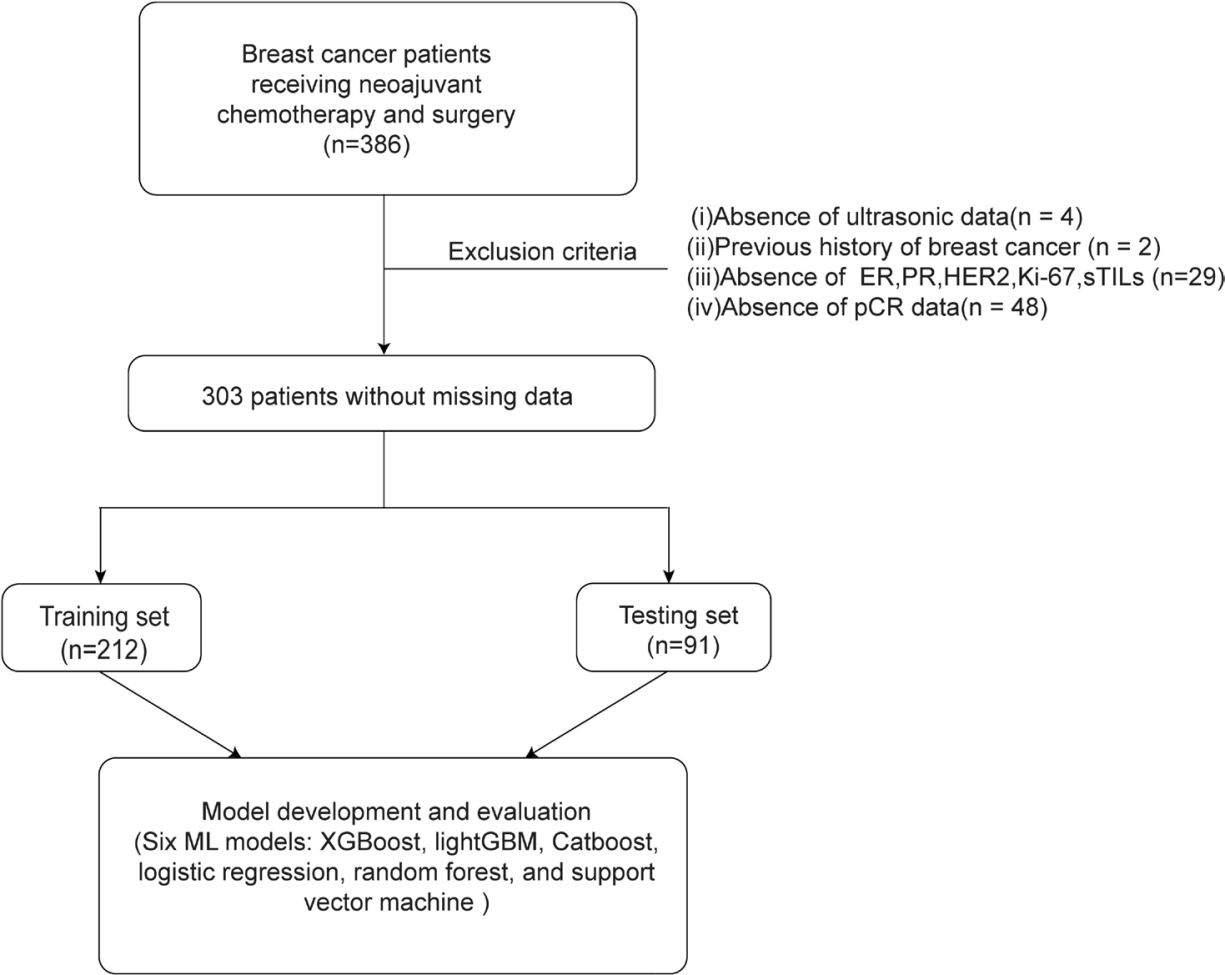
Study flowchart of 386 breast cancer patients treated with NAC and surgery. A total of 303 patients without missing data were incorporated into the final analysis. A Python function was used to randomly divide the dataset into training and testing sets.

This retrospective study analyzed clinicopathological data from 386 BC patients treated with NAC followed by surgery at Zhejiang Provincial People's Hospital between January 2014 and August 2023. Of these, 83 were excluded due to missing data or a history of prior BC, as illustrated in Figure 1. This study received ethical approval from the Ethics Committee at Zhejiang Provincial People's Hospital (Ethics number: 2019KY274). The requirement for informed consent was waived because the data were gathered retrospectively. Meanwhile, the study was designed based on the guidance for reporting clinical prediction models that use machine learning methods (TRIPOD+AI statement) [23].

### 2.2 Predictor Variables

The prediction outcome is determined by the PCR status, which is defined according to the Miller–Payne system following surgery [24]. A total of 22 baseline features were collected from BC patients, including body mass index (BMI), age, menopausal status, red blood cell distribution width (RDW), mean platelet volume (MPV), platelet distribution width (PDW), carcinoembryonic antigen (CEA), and cancer antigens (CA125 and CA153). Tumor size, shape, internal echo, aspect ratio, calcification, posterior echo, abnormal blood flow signal, and lymphatic metastasis were gathered. In addition, pathological markers encompassing Ki‐67 expression, estrogen receptor (ER), progesterone receptor (PR), and human epidermal growth factor receptor 2 (HER2) levels were also sourced from core‐needle biopsy specimens before NAC. Particularly, sTILs were quantified by two pathologists using the baseline core‐needle biopsy specimens.

### 2.3 Data Preprocessing and Feature Selection

Age, BMI, RDW, PDW, MPV, CEA, CA125, and CA153 were treated as continuous variables, while eight ultrasonic features were categorized as categorical variables. ER, PR, and HER2 status were classified into three categories: negative, weakly positive, and strongly positive. Numerical values of Ki‐67 expression and sTILs were analyzed directly to retain all available information. Feature selection was performed through the least absolute shrinkage and selection operator (LASSO) method, which was applied to filter and select predictive variables to prevent overfitting [25]. The process of selecting key clinical factors using LASSO was carried out in the training dataset.

### 2.4 Model Development and Model Evaluation

A stratified random sampling method was employed to divide the dataset into training (70%) and testing (30%) datasets. The training dataset was employed to develop six ML models, and the testing dataset was used to evaluate their prediction performance. Bayesian optimization and grid‐search method were applied to fine‐tune the hyperparameters of the ML models in the training dataset [26]. Each model's performance was evaluated on the testing dataset using the area under the receiver operating characteristic curve (AUC) and decision curve analysis (DCA) to determine the best‐performing model, as well as accuracy, precision, recall, F1 score, and Brier score. The calibration curve was utilized to assess the best‐performing ML model.

### 2.5 ML Explainable Tool

The best‐performing ML model was interpreted using SHapley Additive exPlanations (SHAP), a unified framework for quantifying the contribution and influence of each feature on the ultimate prediction [27]. SHAP values highlighted the positive or negative contribution of each predictor to the target variable. Additionally, SHAP values were utilized to interpret individual cases within the dataset, enhancing the transparency and clinical applicability of the model.

### 2.6 Statistical Analysis

All analyses were conducted using R V4.3.2 and Python V3.7.0. Categorical variables were reported as total numbers and percentages, and group comparisons were made using either chi‐square tests or Fisher's exact test. Continuous variables were expressed as the mean ± standard deviation for normally distributed data or as the median and interquartile range (median, IQR) for skewed distributions. Group differences were evaluated using the *t*‐test for normally distributed data and the Mann–Whitney *U* test for skewed data. Statistical significance was set at *p* < 0.05.

Six ML models—light gradient boosting machine (LightGBM), eXtreme gradient boosting (XGBoost), categorical boosting (CatBoost), logistic regression (LR), random forest (RF), and support vector machine (SVM)—were developed for pCR prediction. Model performance was compared using receiver operating characteristic (ROC) curves and decision curve analysis (DCA). The SHAP framework was employed to interpret the importance of features in the best‐performing model.

## 3 Results

### 3.1 Patient Characteristics

A total of 386 bc patients were retrospectively included in this study. The patient screening process and the study design flow chart are presented in Figure 1. After exclusions, 303 patients without missing information were included in the final ML analysis. Among these were 89 patients (29.37%) who achieved pCR following NAC. The mean age of the cohort was 49.45 years, and the mean BMI was 23.83. Differences between the training and testing datasets are presented in Table 1. No major differences were observed between the training and testing datasets.

**TABLE 1:** Patient features.

Features	*N* = 303	Training dataset (*n* = 212)	Testing dataset (*n* = 91)	*p*
Age	49.45 ± 10.45	49.97 ± 10.66	48.24 ± 9.88	0.2
BMI	23.83 ± 3.93	23.62 ± 3.45	24.30 ± 4.86	0.2
RDW	13.15 ± 1.58	13.11 ± 1.45	13.26 ± 1.84	0.5
PDW	13.67 ± 2.82	13.63 ± 2.67	13.77 ± 3.16	0.7
MPV	10.99 ± 1.23	10.97 ± 1.16	11.03 ± 1.39	0.7
CEA	2.0 (1.3, 3.2)	2.1 (1.4, 3.3)	1.8 (1.3, 2.7)	0.08
CA125	14 (10, 20)	14 (10, 20)	15 (10, 20)	0.8
CA153	18 (12, 26)	18 (13, 27)	15 (11, 25)	0.07
Ki‐67 expression (%)	30 (18, 50)	30 (20, 50)	30 (15, 43)	0.2
sTILs (%)	20 (10, 30)	20 (10, 30)	20 (15, 30)	0.04
Menopausal				0.04
Premenopausal	162 (53%)	105 (50%)	57 (63%)	
Postmenopausal	141 (47%)	107 (50%)	34 (37%)	
Tumor size (US)				0.2
≤ 2 cm	48 (16%)	31 (15%)	17 (19%)	
2–5 cm	196 (65%)	138 (65%)	58 (64%)	
≥ 5 cm	50 (17%)	39 (18%)	11 (12%)	
Trespass chest wall/skin	9 (3.0%)	4 (1.9%)	5 (5.5%)	
Tumor shape (US)				0.7
Regular	11 (3.6%)	7 (3.3%)	4 (4.4%)	
Irregular	292 (96%)	205 (97%)	87 (96%)	
Tumor internal echo (US)				0.1
Uniform	22 (7.3%)	19 (9.0%)	3 (3.3%)	
Uneven	281 (93%)	193 (91%)	88 (97%)	
Aspect ratio (US)				0.1
Balance	281 (93%)	200 (94%)	81 (89%)	
Imbalance	22 (7.3%)	12 (5.7%)	10 (11%)	
Calcification (US)				0.2
Negative	126 (42%)	83 (39%)	43 (47%)	
Positive	177 (58%)	129 (61%)	48 (53%)	
Posterior echo (US)				0.8
Attenuation	83 (27%)	56 (26%)	27 (30%)	
Unchanged	214 (71%)	152 (72%)	62 (68%)	
Enhancement	6 (2.0%)	4 (1.9%)	2 (2.2%)	
Abnormal blood flow signal (US)				0.2
Negative	50 (17%)	31 (15%)	19 (21%)	
Positive	253 (83%)	181 (85%)	72 (79%)	
Lymphatic metastasis (US)				0.01
Negative	119 (39%)	73 (34%)	46 (51%)	
Positive	184 (61%)	139 (66%)	45 (49%)	
ER				0.4
Negative	135 (45%)	92 (43%)	43 (47%)	
Weakly positive	36 (12%)	23 (11%)	13 (14%)	
Strongly positive	132 (44%)	97 (46%)	35 (38%)	
PR				0.3
Negative	143 (47%)	98 (46%)	45 (49%)	
Weakly positive	83 (27%)	55 (26%)	28 (31%)	
Strongly positive	77 (25%)	59 (28%)	18 (20%)	
HER2				0.04
Negative	63 (21%)	36 (17%)	27 (30%)	
Weakly positive	108 (36%)	79 (37%)	29 (32%)	
Strongly positive	132 (44%)	97 (46%)	35 (38%)	
pCR	89 (29%)	65 (31%)	24 (26%)	0.5

*Note:* Data format: *x* ± *s*, Median (IQR); *n* (%). Training set and Testing set were separated by Python function: train_test_split. The 22 features were tested simultaneously, the *p*‐value threshold was corrected by Bonferroni correction, and the statistical significance was set at *p*‐value < 0.0023 (0.05/22).

Abbreviations: BMI, body mass index; ER, estrogen receptor; HER2, human epidermal growth factor receptor‐2; Ki‐67, Ki‐67 expression (%); MPV, mean platelet volume; PDW, platelet distribution width; PR, progesterone receptor; RDW, red blood cell distribution width; sTILs, stromal tumor infiltrating lymphocytes; US, ultrasound.

### 3.2 Feature Selection

**FIGURE 2:**
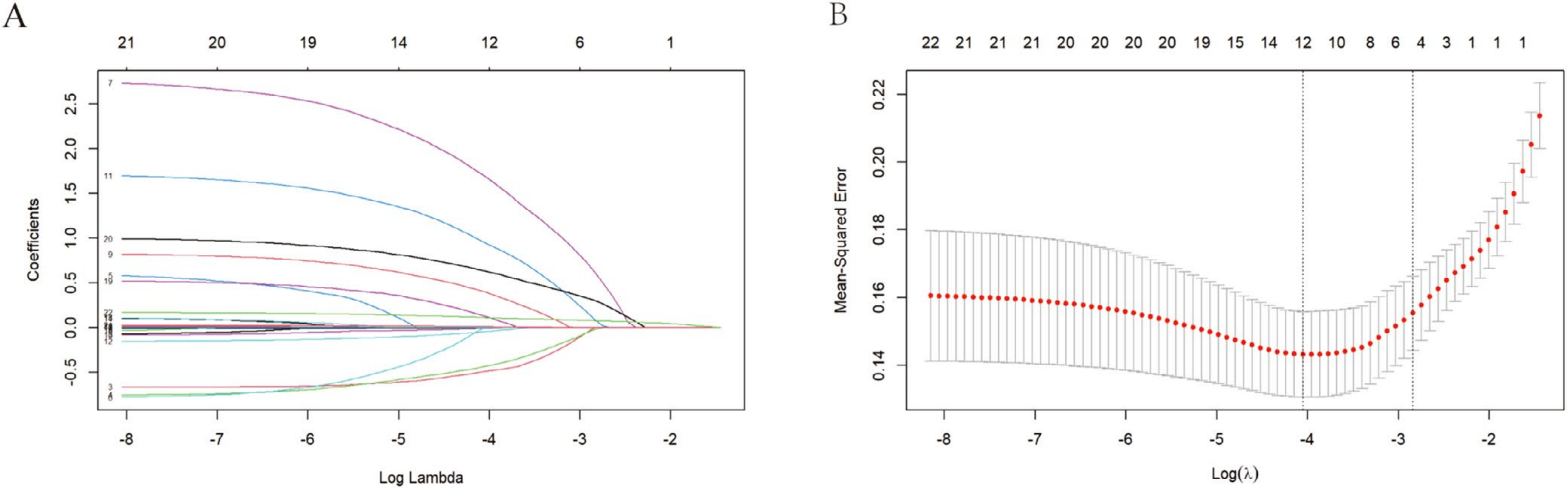
Feature selection using the LASSO binary logistic regression model. (A) LASSO coefficient profiles of 22 features in the training set. The trajectory of the coefficient of each feature was tracked in the LASSO coefficient profiles as lambda varied in the LASSO algorithm. (B) Identification of the optimal penalization coefficient lambda (λ) in the LASSO model in the training set with threefold cross‐validation.

A correlation analysis was performed on the 22 predictive features of the model; the results demonstrated no strong correlation (Figure S1). Features were automatically recognized in the training set using LASSO regression (Figure 2). LASSO regression effectively prevents overfitting and reduces the loss function by adjusting the regularization coefficient, lambda (*λ*). Twelve of 22 features were chosen with nonzero coefficients in the LASSO regression analysis, which were identified at a shrinkage parameter (lambda.min) of 0.01737013. These 12 features were used to construct the ML models, including baseline age, menopausal status, tumor size, aspect ratio, posterior echo, lymphatic metastasis, RDW, PDW, PR, HER2 status, Ki‐67 expression, and sTILs.

### 3.3 Model Building and Evaluation

**FIGURE 3:**
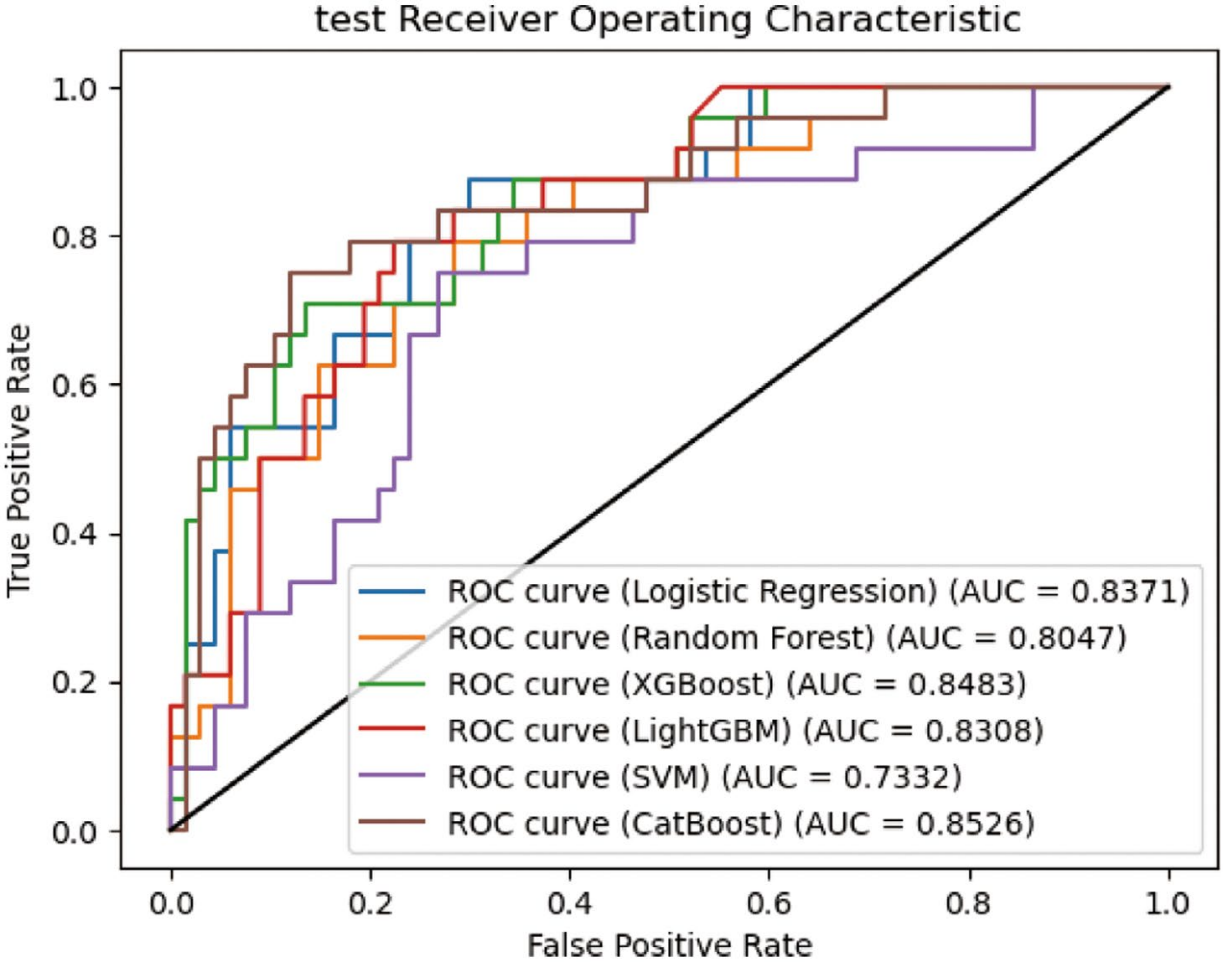
ROC curve for pCR prediction of six ML models in the testing set. CatBoost prediction model (AUC = 0.913).

**FIGURE 4:**
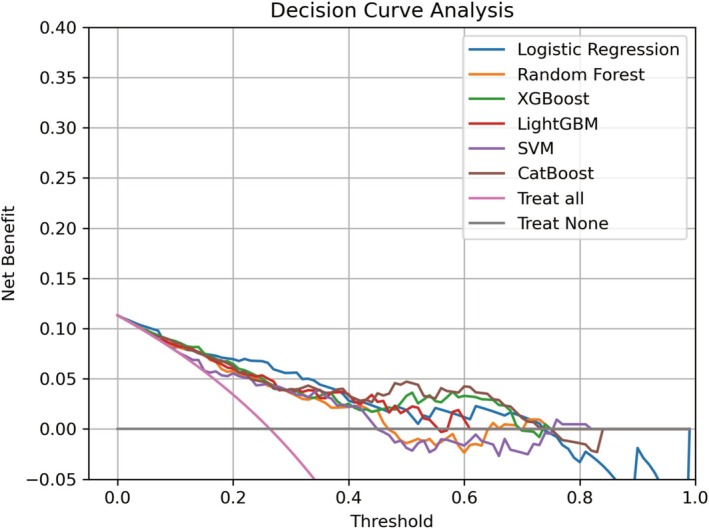
Decision curve analysis (DCA) for six ML models in the testing sets.

**FIGURE 5:**
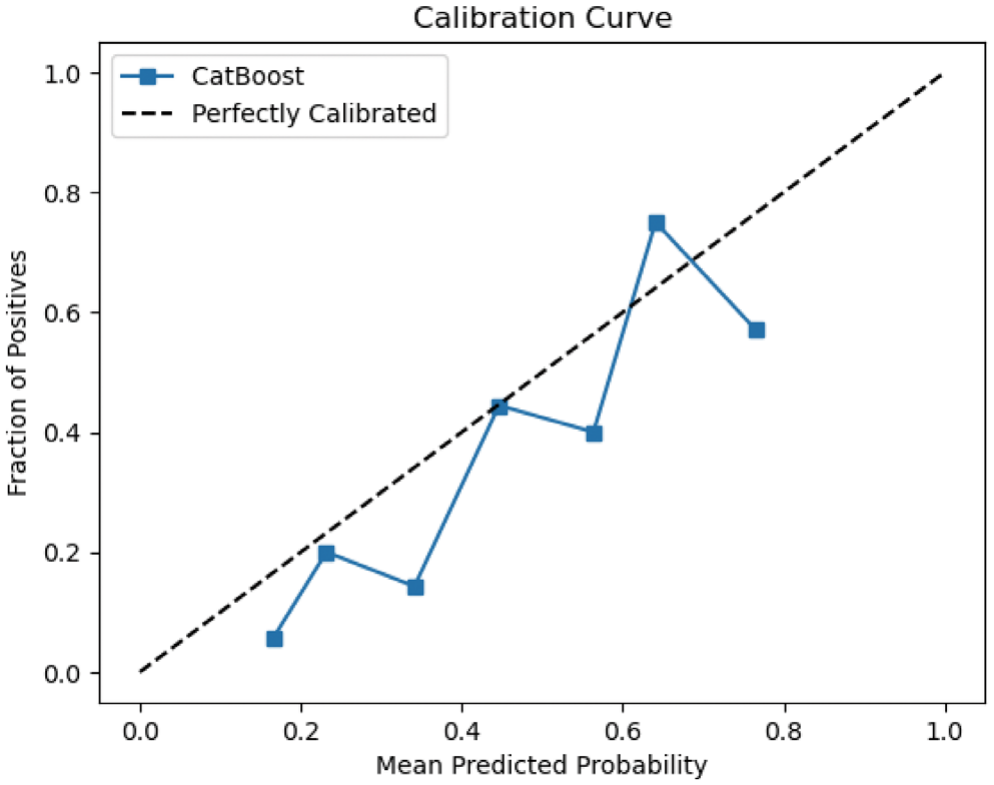
Calibration curve of CatBoost.

We developed six prevalent ML models—XGBoost, LightGBM, CatBoost, LR, RF, and SVM—aimed at predicting pathological complete response to NAC in BC patients. In the training dataset, hyperparameter tuning was realized by Bayesian optimization and grid‐search method. The performance of six ML models was subsequently evaluated employing the testing dataset. The AUC values of XGBoost, LightGBM, CatBoost, LR, RF, and SVM for the testing dataset were 0.848, 0.831, 0.853, 0.837, 0.805, and 0.733, respectively (Figure 3). Additionally, to fully assess the performance, accuracy, precision, recall, F1 score, and Brier score for all six models are provided in Table 2. Detailed hyperparameters for each ML model are listed in Table 3. Among these models, CatBoost demonstrated the highest predictive performance (AUC = 0.853); its accuracy, precision, recall, F1 score, and Brier score were 0.846, 0.692, 0.750, 0.720, and 0.141, respectively. DCA was performed to assess the clinical utility of the models (Figure 4). According to the DCA plot, using treatment strategies based on any ML model resulted in a greater net benefit than default strategies of treating either all or no patients. At a threshold probability of 50%, the net benefit of the CatBoost model surpassed that of the other five models. Both AUC, precision, recall, F1 score, Brier score, and DCA analyses indicated that the CatBoost had the strongest clinical utility. Based on these findings, CatBoost was selected as the best‐performing model for subsequent analysis. The calibration plots for the CatBoost model are presented in Figure 5. The calibration curve's proximity to the diagonal line shows that the model's predicted probabilities align well with actual proportions, indicating strong calibration.

**TABLE 2:** Performance of different ML models.

Models	Auc	Accuracy	Precision	Recall	F1 score	Brier score
CatBoost	0.852612	0.846154	0.692308	0.75	0.72	0.141
LightGBM	0.830846	0.78022	0.6	0.5	0.545455	0.154
Logistic regression	0.837065	0.78022	0.576923	0.625	0.6	0.146
Random forest	0.804726	0.758242	0.535714	0.625	0.576923	0.159
SVM	0.733209	0.692308	0.416667	0.416667	0.416667	0.175
XGBoost	0.848259	0.813187	0.64	0.666667	0.653061	0.147

**TABLE 3:** ML optimal parameter tuning.

Models	Optimal parameter
Random Forest Classifier	max_depth = 7, max_features = 6, *n*_estimators = 78, min_samples_leaf = 3, min_samples_split = 6
CatBoost Classifier	depth = 3, learning_rate = 0.06, l2_leaf_reg = 4, iterations = 143
XGBClassifier (XGBoost)	*n*_estimators = 294, learning_rate = 0.03, max_depth = 2, colsample_bytree = 0.8, alpha = 1, gamma = 1
SVC (SVM)	kernel = “rbf”, C = 1, gamma = 0.01, probability = True
LGBMClassifier (LightGBM)	*n*_estimators = 143, learning_rate = 0.01, max_depth = 3
Logistic Regression	*C* = 10, max_iter = 50, solver = “liblinear”

**FIGURE 6:**
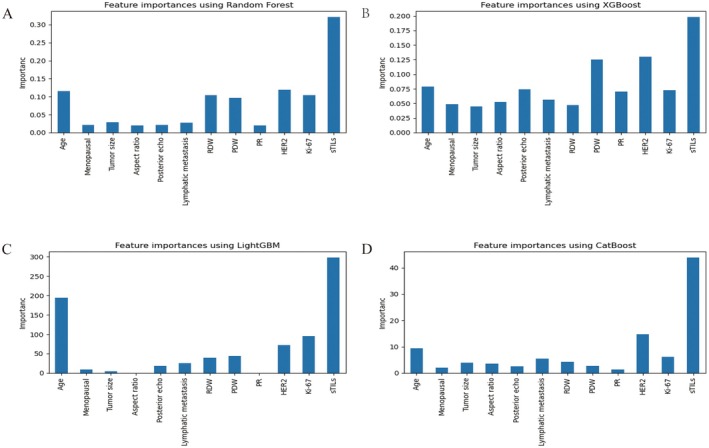
Importance ranking of features in four prediction algorithms. (A) Random Forest, (B) XGBoost, (C) LightGBM, and (D) CatBoost.

Feature importance rankings for four ML models during the six prediction models are shown in Figure 6, including (A) RF, (B) XGBoost, (C) LightGBM, and (D) CatBoost. The importance rankings were derived using built‐in attributes of each ML algorithm. Across these models, sTILs, HER2, and age consistently ranked among the top five predictors most associated with pCR.

### 3.4 Explanation of CatBoost Model With the SHAP Method

**FIGURE 7:**
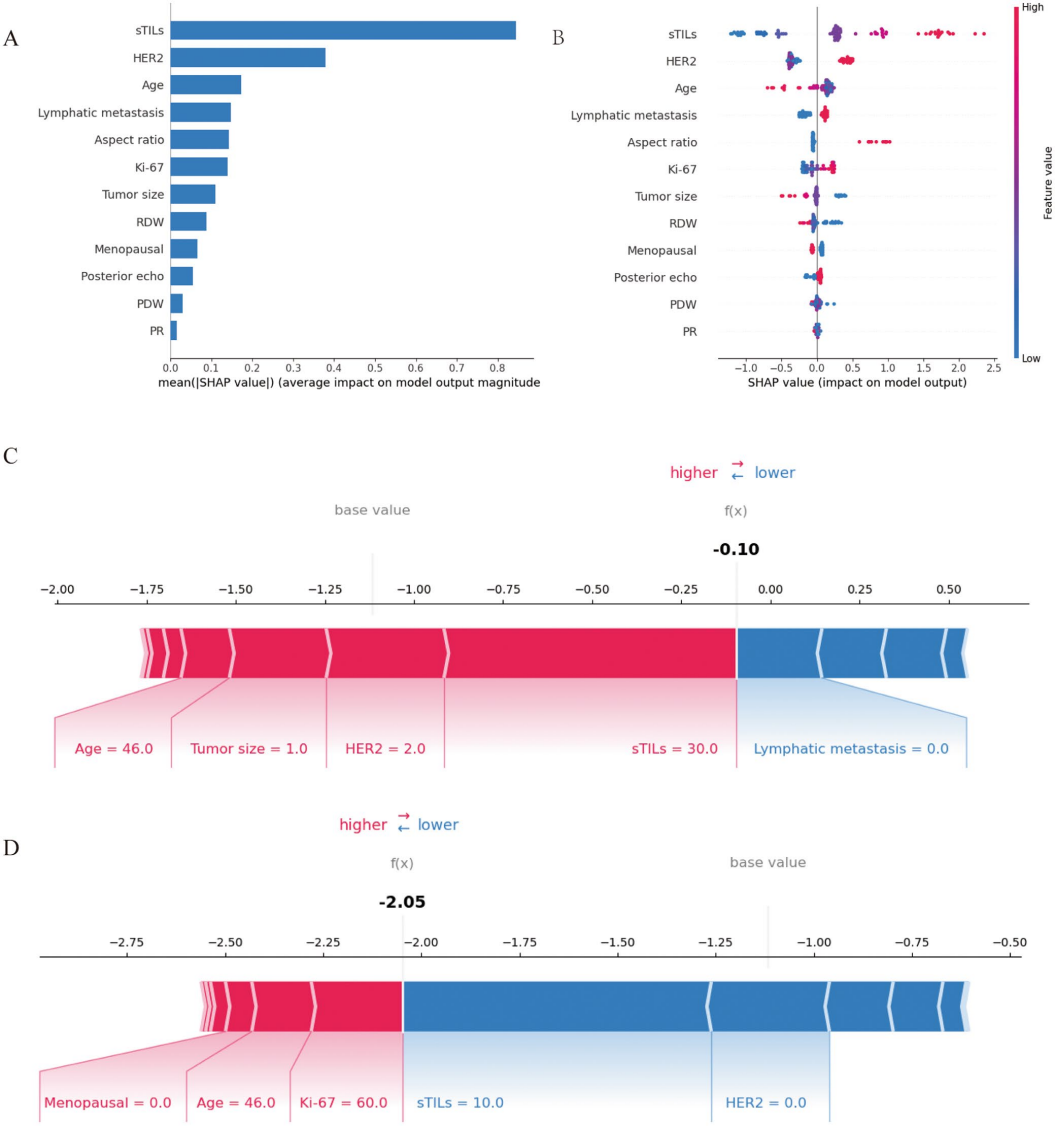
SHAP interpretation of the CatBoost model. (A) Feature importance rankings based on SHAP weights. (B) The effect of each feature on the model output. (C, D) SHAP force plot for individualized predictions of two patients in the testing set, one of whom was predicted to achieve pCR and the other to not achieve pCR. Red bars represent features positively influencing pCR, whereas blue bars represent features negatively influencing pCR. Longer bars indicate greater functional importance of the respective features.

The SHAP algorithm was used to quantify the contribution of each predictor variable to the outcomes predicted by the CatBoost model. The feature importance rankings for the CatBoost model are presented in Figure 7A. STILs demonstrated the highest predictive value, followed by HER2 status, age, lymphatic metastasis, aspect ratio, Ki‐67 expression, tumor size, RDW, menopausal status, posterior echo, PDW, and PR. SHAP values were further employed to identify positive and negative correlations between predictors and the pCR. As illustrated in Figure 7B, the horizontal axis indicates whether a predictor value contributes to a higher (red) or lower (blue) likelihood of pCR. For example, an increase in sTILs positively influenced the prediction, shifting it toward pCR. Similarly, HER2 status and Ki‐67 expression showed positive impacts on pCR prediction. In contrast, an increase in age, tumor size, and RDW negatively influenced the prediction, shifting it toward non‐pCR.

### 3.5 SHAP Individual Force Plots

This study provided examples of individual predictions using SHAP force plots, where red bars represent features that this patient had sTILs at 30%, HER2 status strongly positive (label 2), tumor size ≤ 2 cm (label 1), and an age of 46 years. These features collectively increased the likelihood of achieving pCR as shown in Figure 7C. Conversely, Figure 7D illustrated a patient who did not achieve pCR. This patient had a sTILs level of 10%, Ki‐67 expression of 60%, and HER2 status negative (label 0). These features, particularly the lower sTILs and HER2 status, contributed to a decreased likelihood of pCR.

### 3.6 Importance of sTILs

**FIGURE 8:**
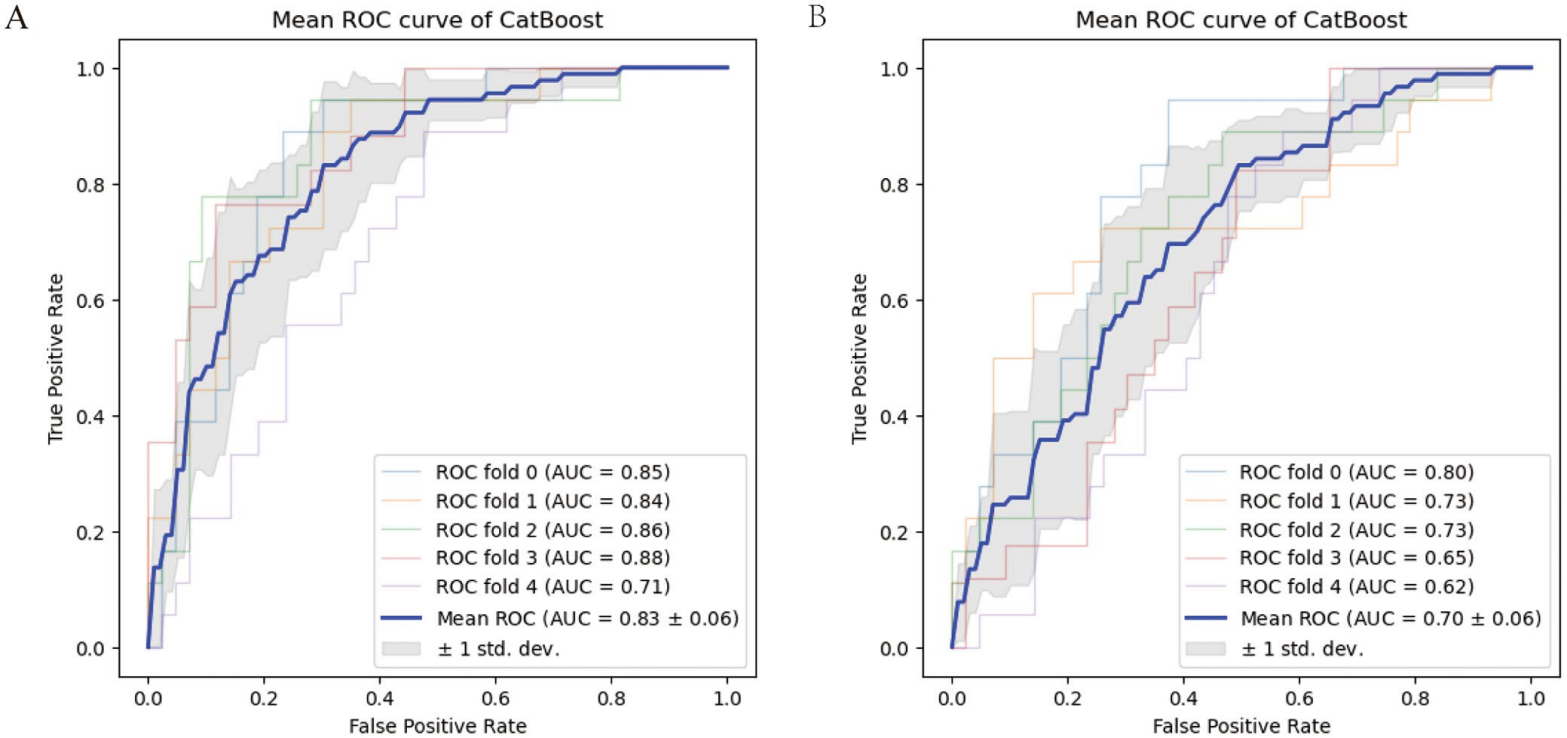
Importance of sTILs. The average AUC of the CatBoost model with a fivefold cross‐validation containing sTILs was 0.83, and the average AUC of the CatBoost model with a fivefold cross‐validation without sTILs was 0.7.

As the sTILs had the strongest predictive value, fivefold cross‐validation was applied to create and confirm the performance of the CatBoost model with or without sTILs in the entire data. The CatBoost model with sTILs had an average AUC of 0.83, as presented in Figure 8A, whereas the average AUC of the CatBoost model without sTILs decreased to 0.70, as shown in Figure 8B. Thus, the sTILs sourced from core‐needle biopsy specimens before NAC had substantial predictive value in the ML model. The Youden's Index of sTILs was 17.5%. A higher value of sTILs indicated that breast cancer patients receiving neoadjuvant chemotherapy were more likely to achieve PCR.

## 4 Discussion

Accurately predicting cancer prognosis is critical for guiding individualized treatment strategies in BC [14, 28, 29]. This study focused on BC patients undergoing NAC, using postoperative pCR as the primary evaluation metric. By analyzing clinicopathological characteristics, predictive factors closely associated with the Miller–Payne grading were identified, and ML prediction models with significant clinical application potential were developed. Based on the actual data features of missing clinical information, we did not perform data interpolation because it involves groups of features from ultrasound exams or biopsy markers, rather than individual features. Finally, a total of 303 BC patients treated with NAC were included, with a pCR rate of 29.37% (89/303). In recent ML studies, Synthetic Minority Over‐sampling Technique (SMOTE) is used to address sample imbalance by generating synthetic samples for the minority class [25]. It is advisable to use SMOTE when the class ratio exceeds 1:10 or 1:20. Model type is important, as some models are more sensitive to imbalance, while decision tree models are less affected. Our study has a 1:3 class ratio and primarily uses tree models, so we analyzed the data directly.

At present, artificial intelligence has been widely applied in various research fields, and AI related studies have shown promise in enhancing accuracy and efficiency in mammographic screening programs, although there are still limitations in clinical work [30]. In our study, the ML‐based pCR prediction model enables more accurate pCR prediction for BC patients, aiding in optimizing treatment strategies. It further validates the broad prospects of AI in the field of clinical applications.

Unlike previous studies on pCR in BC, this study incorporated sTIL as a key feature [9, 10, 11, 31]. Previous studies have reported that the progression of breast tumors is closely related to the tumor immune microenvironment and tumor resistance [32, 33]. As a critical marker of the tumor microenvironment, sTIL level has demonstrated a strong correlation with tumor response to NAC in BC [19, 20, 22, 34, 35]. In this study, the percentage of sTILs was quantified by two pathologists using baseline biopsy specimens examined under a microscope. Based on our current knowledge, this study represents one of the first to develop and validate six ML algorithms with the feature of sTILs for predicting pCR status in BC patients undergoing NAC.

LASSO algorithm was utilized to select and filter features which could effectively prevent overfitting and reduce the loss function by adjusting the regularization coefficient. Twelve of 22 features were chosen automatically in the LASSO regression analysis in the training dataset, including baseline age, menopausal status, tumor size, aspect ratio, posterior echo, lymphatic metastasis, RDW, PDW, PR, HER2 status, Ki‐67 expression, and sTILs. These 12 features were used to construct the six ML models.

Among the six models tested, the CatBoost model demonstrated excellent performance, outperforming LR, RF, SVM, LightGBM, and XGBoost, achieving an AUC of 0.853 in the testing dataset after hyperparameter tuning with Bayesian optimization [36]. Bayesian optimization is recognized for its efficiency in solving black‐box problems and offers significant advantages over traditional methods such as GridSearch and RandomSearch [37]. We rigorously evaluate the performance of our model, and the results from the testing dataset validate its effectiveness. The CatBoost model has high accuracy, precision, recall, F1 score, and low Brier score. Combining precision and recall (0.692/0.75), the overall performance of the model is balanced. Expanding the sample size could improve model performance. Brier score evaluates the variance between expected and actual outcomes, with a lower score indicating a more effective prediction model [38]. DCA further confirmed that the CatBoost model performed optimally within a relevant threshold probability range of approximately 50%. The calibration plots for the CatBoost model, which are in close proximity to the diagonal line, show that the model's predicted probabilities align well. Notably, the AUCs in this study exceeded those reported in similar research [9, 10, 31]. Pu et al. established a nomogram‐derived prediction of pCR in breast cancer patients treated with neoadjuvant chemotherapy with an AUC of 0.758 [9]. Dell'Aquila et al. evaluated four ML algorithms in predicting pCR in BC patients with AUCs of 0.743–0.754 [10]. A study from South Korea found that LightGBM had the highest prediction performance among six models, with an AUC of 0.7845 [31].

The SHAP method was employed to enhance the interpretability of the CatBoost model, ensuring both robust model performance and clinical relevance. To demonstrate the average importance of each feature, the global SHAP value is plotted as a bar chart. By applying SHAP, several key features associated with pCR in BC patients undergoing NAC were identified, then sTILs emerged as the most important predictor, followed by HER2 status, age, lymphatic metastasis, aspect ratio, Ki‐67 expression, tumor size, RDW, menopausal status, posterior echo, PDW, and PR.

Additionally, the CatBoost model incorporating sTILs achieved an average AUC of 0.83 in the fivefold cross‐validation throughout the whole dataset. But the average AUC of the CatBoost model without sTILs decreased to 0.70. This meticulous measurement process demonstrated that sTILs are significant predictors of pCR status, highlighting their value in ML‐based prognostic models.

Furthermore, interpretable ML revealed key relationships between predictive features and pCR outcomes. Higher sTIL levels and Ki‐67 expression were associated with a greater likelihood of achieving pCR following NAC. Conversely, elevated baseline levels of older age, higher RDW, and larger tumor size were linked to a lower probability of achieving pCR. Additionally, attenuation of the aspect ratio and posterior echo in ultrasound imaging was related to a decreased probability of pCR. HER2 strongly and positively influenced pCR, whereas PR and PDW had a weak impact.

This study contributed to a broader comprehension of the relationship between pCR and clinicopathological features in breast cancer. However, several limitations must be recognized. First, the sample size was relatively small, which may constrain the generalizability of our findings. Second, the retrospective nature of this analysis introduced potential biases that could impact the outcomes. These limitations underscored the need for larger studies to validate and extend our findings. A comprehensive trial is anticipated to both corroborate these results and refine the predictive model for more extensive clinical applications moving forward.

## 5 Conclusions

The interpretable CatBoost prediction model developed in this study demonstrated certain predictive performance and clinical significance in forecasting pCR following NAC in BC patients. By leveraging the clinicopathological characteristics of newly diagnosed patients, this model provided a valuable tool for personalized treatment planning and enhancing clinical decision‐making.

## Author Contributions

**Shan Fang:** conceptualization, formal analysis, validation, writing – original draft. **Jun Zhang:** writing – review and editing. **Chengyan Han:** formal analysis. **Mingxiang Kong:** data curation. **Haibo Zhang:** project administration. **Miaochun Zhong:** investigation, funding acquisition. **Wuzhen Chen:** data curation. **Hongjun Yuan:** investigation. **Wenjie Xia:** conceptualization, writing – original draft, funding acquisition. **Wei Zhang:** validation, writing – review and editing.

## Ethics Statement

Ethical approval was granted by the Ethics Committee of Zhejiang Provincial People's Hospital (Approval No. 2019KY274).

## Conflicts of Interest

The authors declare no conflicts of interest.

## Supporting information


**Figure S1:** The correlation heatmap of all features (*p* < 0.8).

## Data Availability

The data that support the findings of this study are available on request from the corresponding author. The data are not publicly available due to privacy or ethical restrictions.
