# Supplementary figures and images for: Interpretable Machine Learning for Predicting Neoadjuvant Chemotherapy Response in Breast Cancer Using the Baseline Clinical and Pathological Characteristics
Source: Cancer Med. 2025 Sep 8;14(17):e71221. doi: 10.1002/cam4.71221 (PMC12415587; doi:10.1002/cam4.71221)

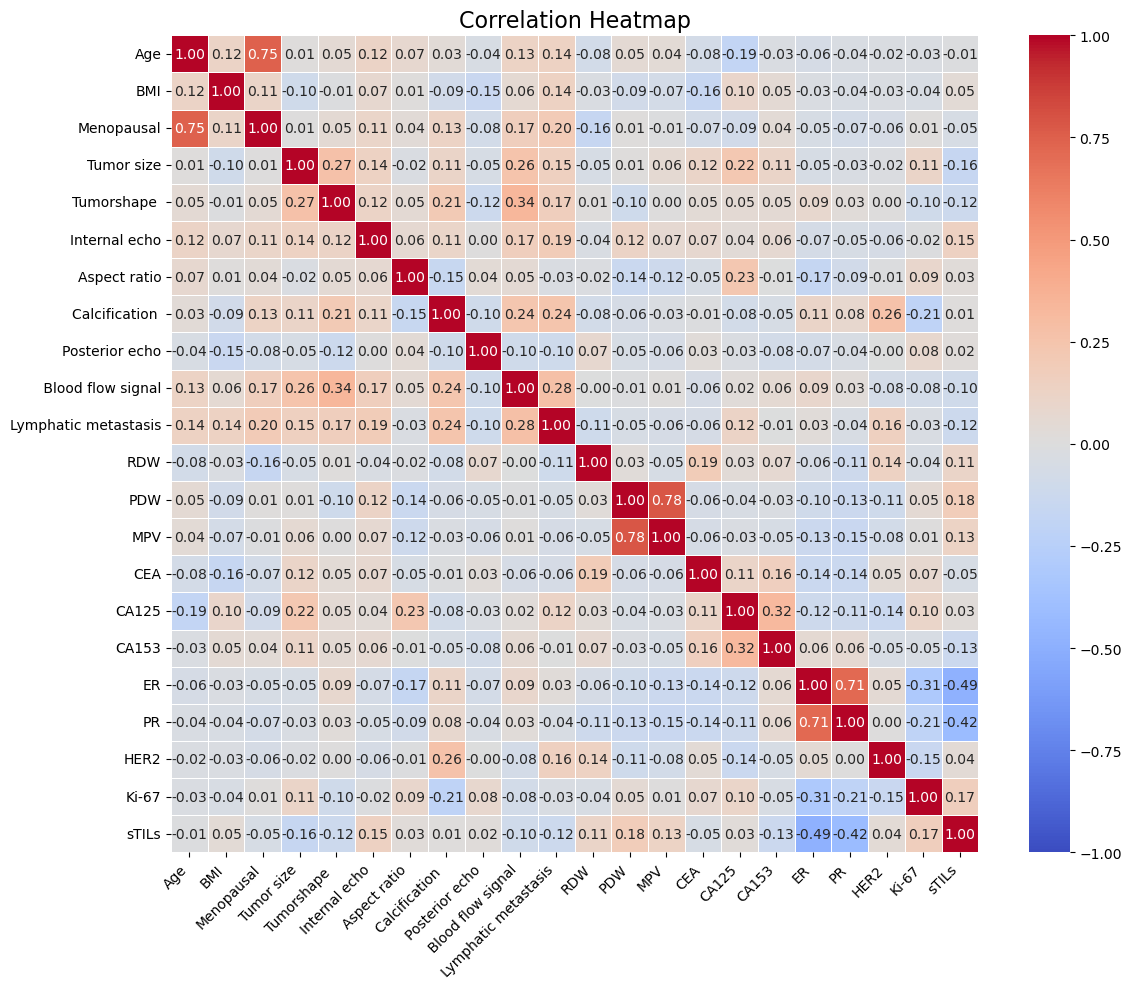


**Figure S1.** The correlation heatmap of all features(p<0.8).

Supplement: Supplementary file 1 — Figure S1: The correlation heatmap of all features (p < 0.8). [file CAM4-14-e71221-s001.docx]
